# SNORD89 promotes stemness phenotype of ovarian cancer cells by regulating Notch1-c-Myc pathway

**DOI:** 10.1186/s12967-019-2005-1

**Published:** 2019-08-08

**Authors:** Wenjing Zhu, Jumin Niu, Miao He, Liwen Zhang, Xuemei Lv, Fangxiao Liu, Longyang Jiang, Jing Zhang, Zhaojin Yu, Lin Zhao, Jia Bi, Yuanyuan Yan, Qian Wei, Hong Huo, Yue Fan, Yuzong Chen, Jian Ding, Minjie Wei

**Affiliations:** 1 Department of Pharmacology, School of Pharmacy, China Medical University, No. 77 Puhe Road, Shenyang North New Area, Shenyang, 110122 Liaoning People’s Republic of China; 2 Department of Pharmacy, Qingdao Municipal Hospital, Qingdao, Shandong China; 3 Shenyang Women’s and Children’s Hospital, Shenyang, Liaoning China; 4 Liaoning Engineering Technology Research Center for the Research, Development and Industrialization of Innovative Peptide Drugs, China Medical University, Shenyang, Liaoning China; 5 Department of Pharmaceutics, School of Pharmacy, China Medical University, Shenyang, Liaoning China; 6 Bioinformatics and Drug Design Group, Department of Pharmacy, National University of Singapore, 18 Science Drive 4, Singapore, 117543 Singapore; 7 Division of Anti-tumor Pharmacology, State Key Laboratory of Drug Research, Shanghai Institute of Materia Medica, Chinese Academy of Sciences, Shanghai, China

**Keywords:** Ovarian cancer stem cells, TCGA, SNORNAs, SNORD89

## Abstract

**Background:**

Ovarian cancer is the leading cause of death in gynecological cancer. Cancer stem cells (CSCs) contribute to the occurrence, progression and resistance. Small nucleolar RNAs (SnoRNAs), a class of small molecule non-coding RNA, involve in the cancer cell stemness and tumorigenesis.

**Methods:**

In this study, we screened out SNORNAs related to ovarian patient’s prognosis by analyzing the data of 379 cases of ovarian cancer patients in the TCGA database, and analyzed the difference of SNORNAs expression between OVCAR-3 (OV) sphere-forming (OS) cells and OV cells. After overexpression or knockdown SNORD89, the expression of Nanog, CD44, and CD133 was measured by qRT-PCR or flow cytometry analysis in OV, CAOV-3 (CA) and OS cells, respectively. CCK-8 assays, plate clone formation assay and soft agar colony formation assay were carried out to evaluate the changes of cell proliferation and self-renewal ability. Scratch migration assay and trans-well invasion analysis were used for assessing the changes of migration and invasion ability.

**Results:**

High expression of SNORD89 indicates the poor prognosis of ovarian cancer patients and was associated with patients’ age, therapy outcome. SNORD89 highly expressed in ovarian cancer stem cells. The overexpression of SNORD89 resulted in the increased stemness markers, S phase cell cycle, cell proliferation, invasion and migration ability in OV and CA cells. Conversely, these phenomena were reversed after SNORD89 silencing in OS cells. Further, we found that SNORD89 could upregulate c-Myc and Notch1 expression in mRNA and protein levels. SNORD89 deteriorates the prognosis of ovarian cancer patients by regulating Notch1-c-Myc pathway to promote cell stemness and acts as an oncogene in ovarian tumorigenesis. Consequently, SNORD89 can be a novel prognostic biomarker and therapeutic target for ovarian cancer.

**Electronic supplementary material:**

The online version of this article (10.1186/s12967-019-2005-1) contains supplementary material, which is available to authorized users.

## Background

Ovarian cancer is the leading cause of death in gynecological cancer, since patients with early stage ovarian cancer do not have symptoms of discomfort and 75% of patients have reached advanced stage (stage III or IV) [1]. It was estimated about 295,414 new cases and 184,799 deaths for ovarian cancer worldwide in 2018 according to the American Cancer Society [2]. Moreover, the treatments for ovarian cancer were limited and the death rates are higher than incidence rates because of the resistance to radiotherapy and chemotherapy in ovarian cancer, especially in advanced stage [3]. Numerous studies indicate that the resistance is related to ovarian cancer stem cells, and many researchers proposed to treat ovarian cancer by targeting tumor stem cells [4–6].

SNORNAs are a class of non-coding RNAs widely distributed in the nucleolus of eukaryotic cells and are mainly classified as box C/D SNORNAs and box H/ACA SNORNAs [7, 8]. They are combined with a set of core proteins to form SNORNP. C/D SNORNAs and H/ACA SNORNAs serve as guides to the 2′-*O*-ribose methylation of rRNAs or small nuclear RNA (snRNAs) and isomerization of uridine residues into pseudouridine, respectively [9–11]. Accumulating evidence has indicated the role of SNORNAs in the occurrence and development of various cancers [12–14]. SNORNAs were once thought to be noise in the process of RNA transcription. However, with further research, they were found to be involved in the process of cancer occurrence and development. Many snoRNAs are highly expressed in tumor cells [15], can be used as candidate diagnostic and prognostic markers of cancers [11, 16–18]. Also, some reports promote that snoRNA is critical for the growth, metastasis and self-renewal of cancer cells [19, 20]. SNORA42 expression was associated with expression of stem cell-core transcription factors in lung tumor-initiating cells (TICs) [21]. It was reported that the expression levels of C/D box SNORNAs in acutemyelogenous leukaemia (AML) patients were highly related to in vivo frequency of leukaemic stem cells [20].

So far, there are few reports about SnoRNAs related to ovarian cancer. Here, we screened out SNORNAs related to ovarian patient’s prognosis by analyzing the data of 379 cases of ovarian cancer patients in the TCGA database, and revealed that high expression of SNORD89 was associated with poor outcomes of ovarian cancer patients and SNORD89 had an important role in the stemness regulation of ovarian cancer cells.

## Materials and methods

### Database analysis

Both the clinical data and the RNA-Seq data in ovarian cancer patients shown here were wholly acquired from TCGA ovarian cancer cohort within the Genomic Data Common (GDC) data portal: https://portal.gdc.cancer.gov/. In total, 379 ovarian cancer samples logged in TCGA had both the clinical data and RNA-Seq data available for analysis. TCGA barcode ID for samples and patients in different data files was used to associate those data tables, and clinical data were matched to the RNA-Seq data. The Edge R package was applied to acquire the RNA expression matrix.

### Cell lines and spheroids culture and transfection

Ovarian epithelial cells (HOSEpiC), Ovarian cancer OVCAR-3 (OV) and CAOV-3 (CA) cells were obtained from the American Type Culture Collection (ATCC) and cultured in RPMI-1640 Medium (HyClone, USA) supplemented with 10% fetal bovine serum (Tian Jin Hao Yang Biological Manufacture CL., LTD, China), 1% Penicillin–Streptomycin Solution (Biosharp Company, China) in a humid atmosphere containing 5% CO2 at 37 °C.

OVCAR-3 spheroids (OS) cells were cultured as our previous report [22]. In Brief, OVCAR-3 cells were cultured in suspension in serum-free DMEM-F12 medium (HyClone, USA) supplemented with growth factors of 20 ng/mL EGF (Peprotech Corporation, USA), 10 ng/mL bFGF (Peprotech Corporation, USA), and 2% B27 (Invitrogen Corporation, USA).

The cells were washed with phosphate buffer saline (PBS) and then transiently transfected with 4 μg SNORD89 overexpression plasmid (Shanghai Genechem Co., LTD, China) or SNORD89 silence plasmid (Shanghai Genechem Co., LTD, China) using Lipofectamine 3000 (Invitrogen, USA) following the manufacturer’s instructions.

### RNA isolation and quantitative real-time PCR (qRT-PCR)

Total RNA samples were extracted from cultured cells using the TRIzol reagent (Tiangen Biotech Company, China) according to the manufacturer’s instructions. cDNAs of SNORDs and mRNA were synthesized from total RNAs by using ReverTra Ace qPCR RT Kit (Toyobo Co., LTD, Japan).

qRT-PCR of SNORD89 and CD133, CD44, Nanog, Notch1, c-Myc was performed with the SYBR qPCR Mix (Toyobo Co., LTD, Japan). 10 μL reaction system was set up according to the manufacturer’s instructions and amplified for 40 cycles. The expression levels of SNORD89 and stemness genes were normalized by U6 and GAPDH. The qRT-PCR was performed on qRT-PCR instrument (Applied Biosystems, USA). Melting curve analysis was performed at the end to validate the specificity of the expected PCR product. Relative expression was calculated using the way of 2^−∆∆Ct^. Three independent samples were prepared for each assay, and each experiment was performed three times. Primer names and primer sequences are listed in the table.

**Table:** 

Primer name	Primer sequence
GAPDH forward	CAGGAGGCATTGCTGATGAT
GAPDH reverse	GAAGGCTGGGGCTCATTT
CD133 forward	GTGGCGTGTGCGGCTATGAC
CD133 reverse	CCAACTCCAACCATGAGGAAGACG
CD44 forward	ACAAGCACAATCCAGGCAACTCC
CD44 reverse	TGGTGTTGTCCTTCCTTGCATTGG
Nanog forward	AATACCTCAGCCTCCAGCAGATG
Nanog reverse	TGCGTCACACCATTGCTATTCTTC
Notch1 forward	CCTGAGGGCTTCAAAGTGTC
Notch1 reverse	CGGAACTTCTTGGTCTCCAG
c-Myc forward	CGACGAGACCTTCATCAAAAAC
c-Myc reverse	CTTCTCTGAGACGAGCTTGG

Quantification of SNORD89 and U6 were performed with a stem-loop real time PCR miRNA kit (Ribobio Co., LTD, China).

### Reverse transcription-polymerase chain reaction (RT-PCR) and agarose gel electrophoresis

cDNAs of SNORDs and mRNA were synthesized from total RNAs by using ReverTra Ace qPCR RT Kit (Toyobo Co., LTD, Japan), and were amplified with the SYBR qPCR Mix (Toyobo Co., LTD, Japan). The amplification reaction was going on for 30 cycles.

0.45 g agarose gel (Gene Company, LTD, China) in 45 mL TAE (0.04 M Tris, 0.02 M acetic acid, 0.002 M EDTA; pH adjusted to 8.5 with acetic acid) plus 4.5 μL nucleic acid dyes (BBI Life Science Corporation, China) was melted by microwave and add to the agarose gel box. After hardening, the samples were added into the gel and run for 30 min at 100 V. Bands were visualized by fluorescence over long wavelength ultraviolet light and photographed by agarose gel-electrophoretic apparatus (Beijing Liuyi Biotechnology Co. LTD, China).

### Flow cytometry

For CD133 expression analysis, cells were digested and suspended with PBS. The cell suspension was incubated with FITC-conjugated antibody against CD133 (1:20, BD Pharmingen, USA) at 4 °C for 30 min in darkness, and washed with cold PBS twice, finally for the determination.

For cell cycle analysis, cells were digested and washed with PBS. The cell suspension was fixed with 3 mL cold ethanol overnight at 4 °C. After that, the cells were incubated with 20μL Rnase A at 37 °C for 30 min, then incubated with 400 μL propidium iodide (PI) at 4 °C for 30 min, and analyzed by Flow Cytometry (ACEA Biosciences Inc., China) with the software “NovoExpress 1.2.5” (ACEA Biosciences Inc., China).

### Cell proliferation assay

The cell proliferation was evaluated by Cell Counting Kit-8 (CCK-8) assay (Dojindo, Kumomoto, Japan). Cells were seeded in 96-well plates at a density of 3000 cells/well. After 24 h, 48 h and 72 h of transfection with SNORD89 overexpression plasmid or silence plasmid, CCK-8 solution (10 μL) was added into each well and incubated for 3 h at 37 °C. The OD value of the reaction solution was measured at 450 nm by an Anthos 2010 microplate reader (Anthos Labtec Instruments GmbH, Austria).

### Wound healing assay/in vitro scratch assay

Cells were cultured into 6-well plates until 70% confluency, and then transfected with SNORD89 OE plasmid. Linear ‘scratches’ were created on the monolayer cells in straight lines with sterile tips. The cells were washed three times with PBS, and added serum free medium. The cells were photographed after 0 h, 24 h and 48 h of incubation under a microscope (Nikon Eclipse TE2000-U, Japan). Wound closure was quantified by Image J software.

### Cell invasion assay

The invasion of cells was measured using transwell plates (8.0 μm pore size, Corning, USA). The OV and CA cells transfected with SNORD89 OE plasmids and OS cells transfected with sh-SNORD89 plasmids were starved overnight, and then suspended in serum free medium. 100 μL single cell suspension with 20000 cells was seeded into the upper chamber precoated with Matrigel, and 600 μL medium with 15% fetal bovine serum was added into the lower chamber. 48 h later, the cells at the upper side were removed, and the cells that invaded to the lower side were fixed and further stained with crystal violet. The number of cells in three random fields was counted for each filter by Image J software.

### Clonogenic assay

The OV and CA cells (1000 cells/well) transfected with SNORD89 OE plasmids were placed in 6-well plates and maintained in medium containing 15% FBS. After 14 days, the cells were fixed and stained by crystal violet. Visible colonies were then counted manually. Each well was assessed in triplicate.

### Soft agar colony formation assay

Soft agar colony formation assay was carried out as described previously [22]. Briefly, 1.2% agarose gel (Lonza Rockland, ME USA) was mixed with 1640 medium containing 20% FBS and 5% Penicillin–Streptomycin solution as a bottom layer in 6-well plates. The OV and CA cells transfected with SNORD89 OE plasmids (5000 cells/well) were mixed into a top layer in 0.6% agarose gel and the same medium. After the incubation for 3 weeks at 37 °C, the colonies were stained with MTT and counted.

### Spheroid formation assay

The OS cells transfected with sh-SNORD89 or sh-NC plasmids were seeded onto Ultra-Low Attachment Surface 6-well plates (Guangzhou Jet Bio-Filtration Co., LTD, China) at a density of 2000 cells/well in serum-free DMEM-F12 medium supplemented with 20 ng/mL EGF, 10 ng/mL bFGF, and 2% B27. Fresh medium of 0.5 mL was added into each well every 3 days. After the culture for 2 weeks in a humid atmosphere containing 5% CO2 at 37 °C, the number of the spheres > 50 μm in diameter was counted under an inverted microscope (Nikon TE2000-U, Japan).

### Western blot

Protein was extracted from OV, CA and OS cells after SNORD89 interference, and subjected to SDS-polyacrylamide separating gel and transferred onto a polyvinylidene fluoride (PVDF) membrane. The primary antibodies used in these experiments were c-Myc (1:1000, Cell Signaling Technology, USA), Notch1 (1:1000, Cell Signaling Technology, USA), β-actin (1:1000, Absin Bioscience Inc, China). The bands were visualized by enhanced chemiluminescence (ECL).

### Statistical analysis

The Log-rank (Mantel–Cox) test was used for survival analysis by GraphPad Prism 7.0. The link between SNORNAs’ expression and clinicopathologic features of ovarian cancer patients was assessed using the Chi square test and unpaired t test by SPSS Statistics 24.0 software. HRs were calculated using the Cox proportional hazard model, and 95% confidence intervals (CI) were also determined by SPSS Statistics 24.0 software. All other analyses were performed with GraphPad prism7.0 using unpaired t test. Differences were considered statistically significant when the P-value was < 0.05.

## Results

### Screening SNORNAs related to prognosis in ovarian cancer patients

**Fig. 1:**
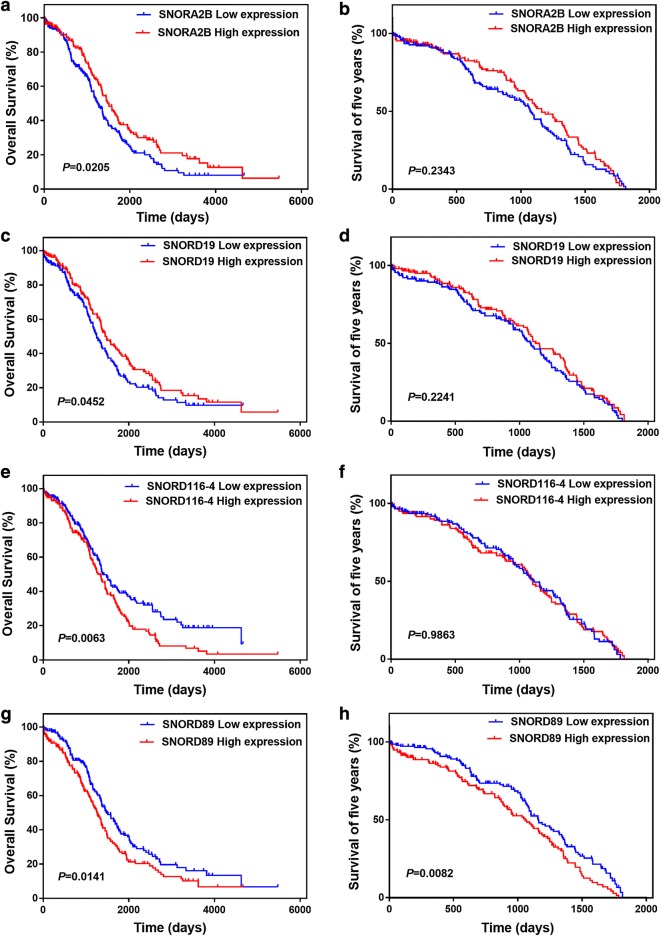
Prognostic impact of snoRNAs expression status in patients with ovarian cancer in TCGA (n = 379). Kaplan–Meier survival curves for overall survival (OS) and five years survival in ovarian cancer patients based on the expression of **a**, **b** SNORA2B, **c**, **d** SNORD19, **e**, **f** SNORD116-4, **g**, **h** SNORD89

To find which SNORNAs are related to poor prognosis of ovarian cancer, we used TCGA database to analyze the relationship between of SNORNAs expression and overall survival for 379 ovarian cancer patients. Finally, 4 SNORNAs (SNORA2B, SNORD19, SNORD116-4 and SNORD89) associated with poor prognosis of ovarian cancer were screened out by Kaplan–Meier analysis. Low expression of SNORA2B and SNORD19 is related to poor prognosis of ovarian cancer patients (Fig. 1a, c), and high expression of SNORD116-4 and SNORD89 is associated with poor prognosis of ovarian cancer patients (Fig. 1e, g). Next, we analyzed these four SNORNAs resort to SNORic database. Based on the retrieval of SNORic database, we found that SNORD89 was correlated with 178 mRNAs and participated in splicing of 43 mRNAs in ovarian cancer. SNORA2B, on the other hand, was correlated with only two mRNAs and not participated in mRNA splicing in ovarian cancer. SNORD19 is involved in splicing of 2 mRNAs and not correlated with any mRNA. Further, there is no information of SNORD116-4 in ovarian cancer in SNORic database.

TO avoid the influence of a small number of patients who survived for more than 5 years on survival curve differences, we set a 5-year cut-off data for survival to observe the effect of screened snoRNAs on patients’ prognosis. We found that only the SNORD89 dysregulation was significantly correlated with poor prognosis of patients (Fig. 1b, d, f, h). Thus, these results suggest that SNORA2B and SNORD19 may play roles as suppressor genes, while SNORD116-4 and SNORD89 as oncogenes in ovarian cancer.

In order to comprehend the correlation between the oncogenes and poor prognosis of ovarian cancer patients, we further selected SNORD89 and SNORD116-4 for our study. The ovarian cancer patients in TCGA database are mainly in stage III and IV. Therefore, we analyzed the relationship between the expression of SNORD89, SNORD116-4 and prognosis of patients in stage III and IV. The survival curves showed that the expression of SNORD89 was an important prognostic factor in stage IV, and patients with high expression of SNORD89 have more poorly prognosis (Additional file 1: Figure S1a, b). Among the ovarian cancer patients in TCGA, 152 and 24 patients had high expression of SNORD116-4 in stage III and stage IV, 139 and 33 patients had low expression of SNORD116-4 in stage III and stage IV, respectively. The patients in stage III with high expression of SNORD116-4 have poor prognosis, however, patients in stage IV with high expression of SNORD116-4 have better prognosis, SNORD116-4 shows a trend for better survival in stage IV (Additional file 1: Figure S1c). These results suggest that SNORD116-4 has different effects on the prognosis of ovarian cancer in stage III and stage IV.

Moreover, we further confirmed that the prognosis of patients in stage IV was worse than that in stage III (Additional file 1: Figure S1d). Interestingly, this was reversed by the SNORD89 high expression. From the survival curve, we can see that the patients with SNORD89 high expression in stage III have worse prognosis than the patients with SNORD89 low expression in stage IV (Additional file 1: Figure S1e). In addition, we analyzed the effects of SNORD89, SNORD116-4 and stage on the 5-year survival of patients with ovarian cancer. Unlike the OS curves, SNORD116-4 had no significant effect on the 5-year survival of patients with stage III and IV, and, stage has a strong correlation with the 5-year survival of patients (Additional file 2: Figure S2). However, the prognosis of patients with high SNORD89 expression was worse than that of patients with low SNORD89 expression in OS and 5-year survival curves. The findings suggest that SNORD89 might have an important role in the progress of ovarian cancer.

### Prognostic factors of ovarian cancer patients

The high expression of SNORD89 and SNORD116-4 predicts poor prognosis in ovarian cancer patients. We next analyzed the correlation between the two SNORNAs expression and the clinicopathologic features of ovarian cancer patients in TCGA database by Chi square test. We found that the expression of SNORD116-4 was not correlated with any clinicopathological parameters. However, the expression of SNORD89 was correlated with age (*P *= 0.03, Additional file 3: Table S1). We also found that the expression of SNORD89 was higher in elderly patients (*P *= 0.0202) and progressive disease (*P *= 0.0486) by unpaired t test (Additional file 4: Figure S3a, b).

In order to observe SNORD89 expression and the clinical factors affect the prognosis of ovarian cancer, the univariate and multivariate COX’s regression analysis were performed (Additional file 3: Tables S2 and S3). Univariate analysis showed that the expression of SNORD89, age, and tumor size was significantly associated with overall survival (OS), and age was also related to progression-free survival (PFS) of ovarian cancer patients. In addition, multivariate COX’s regression analysis revealed that race and venous invasion were independent predictors of OS, and venous invasion was also independent predictors of PFS in patients with ovarian cancer. Thus, SNORD89 expression may be associated with other factors affecting the prognosis of ovarian cancer patients.

### SNORD89 is highly expressed in ovarian cancer stem cells

**Fig. 2:**
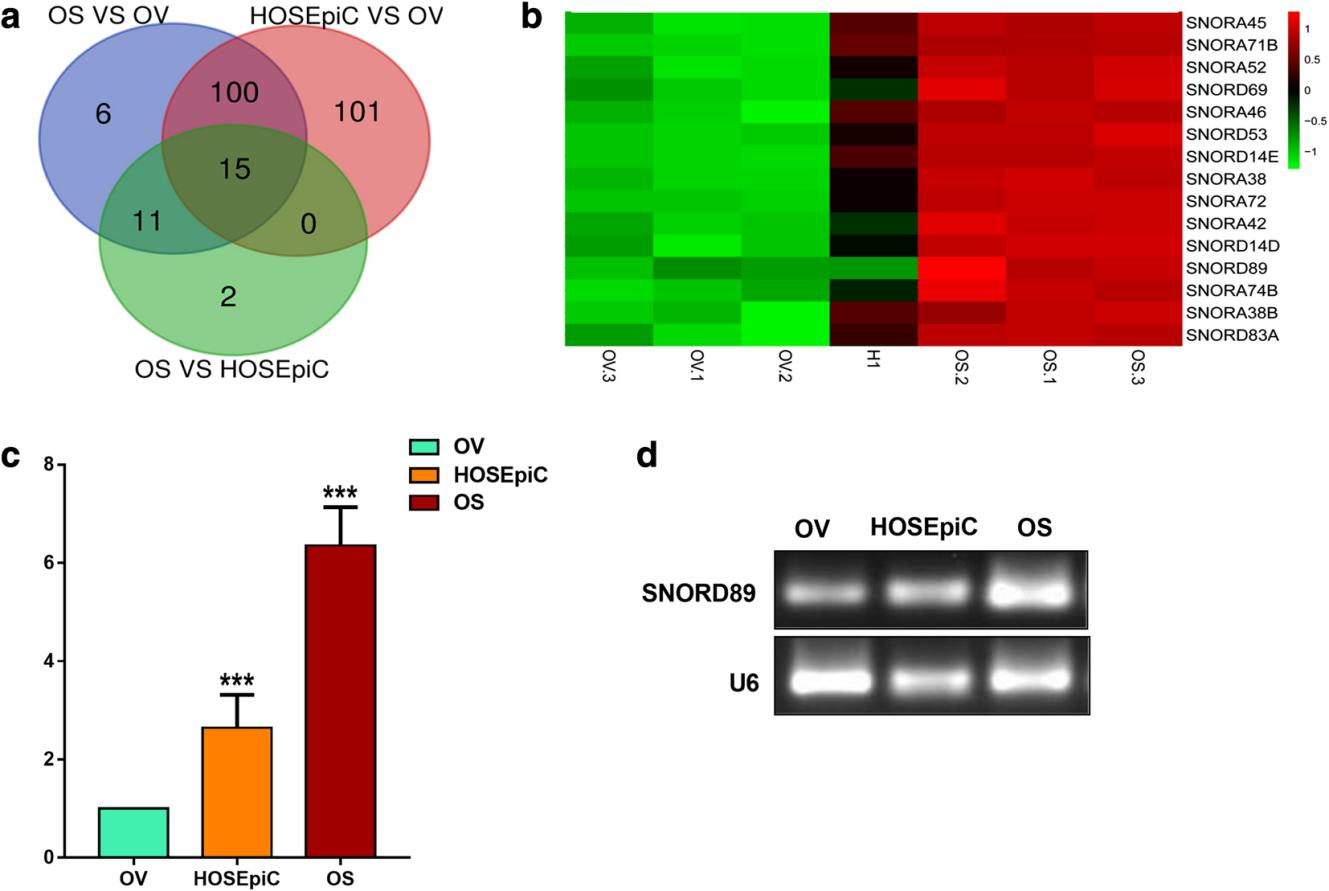
SNORD89 is highly expressed in ovarian cancer stem cells. **a**, **b** 15 up-regulated snoRNAs were screened out in HOSEpiC and OS cells by genes chip. **c** The SNORD89 expression was analyzed in OV, HOSEpiC and OS cells by qRT-PCR. The expression of SNORD89 in OV cells was set as 1. **d** The representative agarose gel electrophoresis showed the increased SNORD89 expression in HOSEpiC and OS cells. U6 was used as an endogenous control

Tumor stem cells cause tumor recurrence by promoting tumor invasion, metastasis, drug resistance, and so on, leading to poor prognosis of patients [23]. It has been reported that SNORNAs have important roles in the cancer progress [12]. We compared the non-coding RNAs (NC RNAs) expression in ovarian epithelial cells (HOSEpiC), ovarian cancer cells (Ovcar-3, OV) and ovarian cancer stem cells (Ovcar-3-S, OS) by gene chips. Finally, 15 SNORNAs with high expression in HOSEpiC and OS were screened out (Fig. 2a, b, Additional file 3: Table S4). Among them, only SNORD89 and SNORD116-4 were associated with the prognosis of ovarian cancer patients, while SNORD116-4 played an opposite role in stage III and stage IV. Therefore, SNORD89 can better predict the prognosis of ovarian cancer patients. As we know, the poor prognosis of tumors is closely related to cancer stem cells. To our surprise, SNORD89 is not only associated with poor prognosis of ovarian cancer, but also highly expressed in OS vs OV and OS vs HOSEpiC, while SNORD116-4 was not highly expressed in OS. Therefore, we decided to conduct a further research on SNORD89.

Next, we measured the expression difference of SNORD89 in HOSEpiC, OV and OS cells by quantitative real-time PCR (qRT-PCR) and reverse transcription PCR (RT-PCR). qRT-PCR results showed that SNORD89 expression was up-regulated 2.38 ± 0.29-fold in OS vs HOSEpiC cells, and 5.93 ± 0.53-fold in OS vs OV cells (Fig. 2c). And the increased expression of SNORD89 was also observed by RT-PCR (Fig. 2d). The data suggest that SNORD89 upregulated in OS versus OV and HOSEpiC.

### Effects of SNORD89 interference on the stemness of ovarian cancer cells

**Fig. 3:**
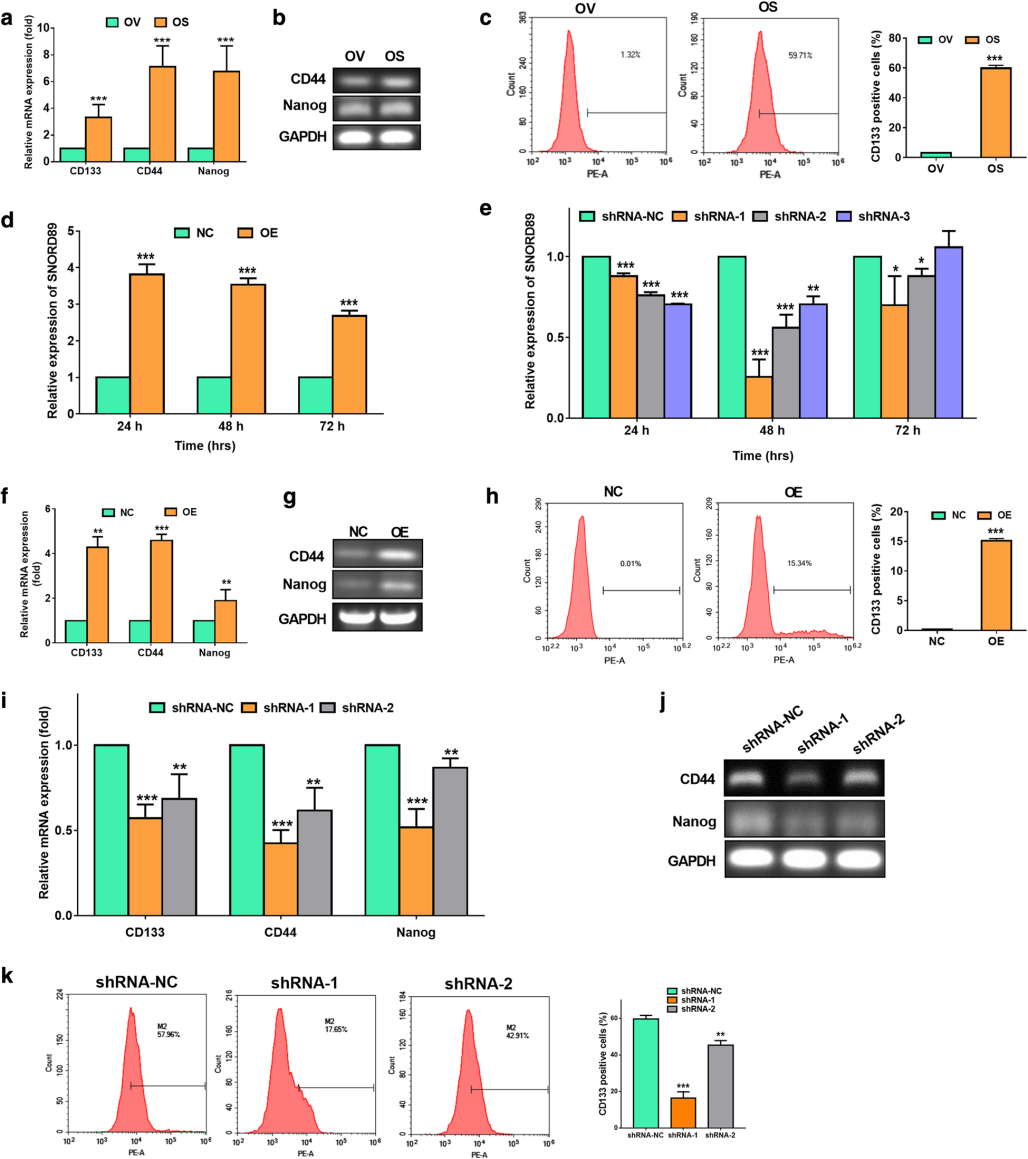
Effects of SNORD89 interference on stemness genes expression in ovarian cancer cells. **a** The mRNA expression of CD133, CD44 and Nanog were detected in OV and OS cells by qRT-PCR. The mRNA expression of these genes in OV cells was set as 1. **b** The representative agarose gel electrophoresis photos showed the expression of CD24 and Nanog in OV and OS cells after RT-PCR. GAPDH was used an endogenous control. **c** The comparison of CD133 positive cells in OV and OS cells by flow cytometry analysis. **d** The SNORD89 expression in OV cells transfected with over expression (OE) of SNORD89 plasmid or negative control (NC) plasmid at 24, 48, and 72 h by qRT-PCR. The SNORD89 expression in OV cells transfected with NC plasmids was set as 1. **e** The SNORD89 expression in OS cells transfected with silence plasmids of SNORD89 (shRNA-1, shRNA-2, shRNA-3) or shRNA negative control (shRNA-NC) plasmid at 24, 48, and 72 h by qRT-PCR. The SNORD89 expression in OS cells transfected with shRNA-NC plasmids was set as 1. **f** The mRNA expression of CD133, CD44 and Nanog were detected in OV cells transfected with SNORD89 OE or NC plasmids at 24 h by qRT-PCR. The mRNA expression of these genes in OV cells transfected with NC plasmids was set as 1. **g** The representative agarose gel electrophoresis photos showed the increased expression of CD44 and Nanog in OV cells transfected with SNORD89 OE. **h** The increased CD133 positive cells in OV cells transfected with SNORD89 OE by flow cytometry analysis. **i** The mRNA expression of CD133, CD44 and Nanog were detected in OS cells transfected with shRNA-1, shRNA-2, and shRNA-NC plasmids at 48 h by qRT-PCR. The mRNA expression of these genes in OS cells transfected with shRNA-NC plasmids was set as 1. **j** The representative agarose gel electrophoresis photos showed the decreased expression of CD44 and Nanog in OS cells transfected with SNORD89 shRNA-1 and shRNA-2. **k** The decreased CD133 in OS cells after silencing SNORD89 by flow cytometry analysis

We compared the expression of genes associated with stemness in OV and OS by qRT-PCR. The expression levels of CD133, CD44, and Nanog were increased by 3.32 ± 0.48, 7.11 ± 0.90 and 6.78 ± 1.10-fold in OS cells relative to OV cells, respectively (Fig. 3a). Also, RT-PCR and agarose gel electrophoresis showed the higher exprssion of CD44 and Nanog in OS cells (Fig. 3b). Additionally, we observed that the CD133 positive cells were significantly increased in OS than those in OV by flow cytometry analysis (Fig. 3c).

To investigate whether the SNORD89 interference affects the stemness of ovarian cancer cells, we first examined the SNORD89 expression in OV cells transfected with over expression plasmid (OE) of SNORD89 and in OS cells transfected with silent plasmid (shRNA) of SNORD89 for 24, 48, and 72 h, respectively. The qRT-PCR analysis showed that the SNORD89 overexpression in OV and CA cells was the most efficient at 24 h’ transfection (3.81 ± 0.28-fold than NC in OV cells and 20.65 ± 1.195-fold than NC in CA cells, Fig. 3d and Additional file 5: Figure S4a). And the most silence efficiency of three shRNA (shRNA-1, shRNA-2, shRNA-3) was 0.255 ± 0.08, 0.56 ± 0.05 and 0.70 ± 0.04 at 48 h in OS cells (Fig. 3e).

Next, we examined the changes of stemness genes in OE-transfected OV cells at 24 h, and in shRNA-transfected OS cells at 48 h. We found that the expression of CD133, CD44, and Nanog was increased by 4.28 ± 0.48, 4.58 ± 0.28 and 1.90 ± 0.49-fold in OE group than NC group in OV cells by qRT-PCR (Fig. 3f), and increased by 1.80 ± 0.25, 1.40 ± 0.11 and 1.47 ± 0.09-fold in OE group than NC group in CA cells by qRT-PCR (Additional file 5: Figure S4b). RT-PCR and agarose gel electrophoresis also verified the elevated expression of CD44 and Nanog (Fig. 3g). The flow cytometry analysis showed the higher CD133 positive cells in OE group (Fig. 3h). Consistantly, the silence of SNORD89 with shRNA-1 and shRNA-2 at 48 h notably decreased the expression of CD44 and Nanog in OS cells by qRT-PCR (Fig. 3i) and RT-PCR and agarose gel electrophoresis (Fig. 3j). The flow cytometry analysis showed the decreased CD133 in OS cells transfected with SNORD89 silence plasmids (Fig. 3k). These data suggest that SNORD89 can increase the expression of stemness genes in ovarian cancer cells.

### Effects of SNORD89 interference on cell proliferation and self-renewal ability of ovarian cancer cells

**Fig. 4:**
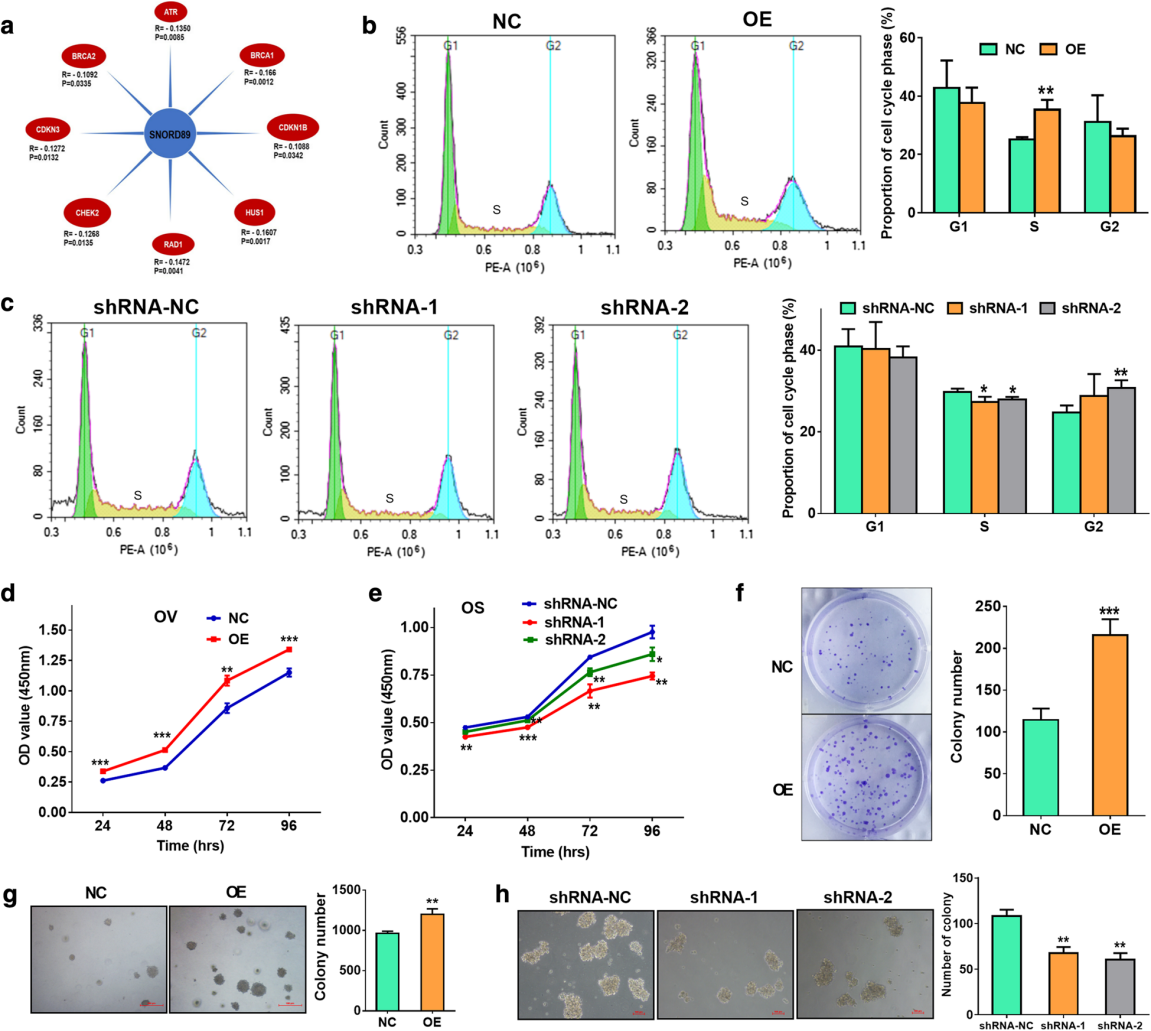
Effects of SNORD89 interference on cell proliferation and self-renewal ability of ovarian cancer cells. **a** The correlation analysis of SNORD89 expression with other genes expression using the data from ovarian cancer patients in TCGA. **b** The flow cytometry analysis of the proportion of cell cycle phases in OV cells transfected with SNORD89 OE plasmids. **c** The flow cytometry analysis of the proportion of cell cycle phases in OS cells transfected with shRNA-1 and shRNA-2 plasmids. **d** The cell proliferation was measured in OV cells of SNORD89 overexpression by Cell Counting Kit-8 (CCK-8) assays at 24 h, 48 h, 72 h and 96 h transfection. **e** The cell proliferation was measured in OS cells of SNORD89 silence by CCK-8 assays at 24 h, 48 h, 72 h and 96 h transfection. **f** The cell proliferation ability was measured in OV cells of SNORD89 overexpression by plate clone formation assay. **g** The cell self-renewal ability was measured in OV cells of SNORD89 overexpression by soft agar colony formation assay. **h** The cell self-renewal ability was measured in OS cells of SNORD89 silence by colony formation assays

We analyzed the expression correlation of SNORD89 with other genes using the data from ovarian cancer patients in TCGA, and found that the expression level of SNORD89 was related to some genes of cell cycle checkpoint and cell cycle arrest (Fig. 4a). So, we suspected that the interference of SNORD89 expression may affect cell cycle. And we examined the changes of cell cycle phases in OV and OS cells after interfering the expression of SNORD89. The flow cytometry analysis showed the proportion of S phase was obviously increased in OV cells transfected with SNORD89 OE plasmids (Fig. 4b). Conversely, the proportion of S phase was obviously decreased in OS cells transfected with shRNA-1 and shRNA-2, while the proportion of G_2_ phase was raised (Fig. 4c). These results indicated that SNORD89 may have an effect on cell proliferation in ovarian cancer.

Then, we checked the cell proliferation ability changes in OV and CA cells of SNORD89 overexpression and OS cells of SNORD89 silence by Cell Counting Kit-8 (CCK-8) assays at 24 h, 48 h, 72 h and 96 h transfection. CCK-8 assays revealed a significant raise cell proliferation in OE cells compared with the NC cells (Fig. 4d and Additional file 5: Figure S4c), and a notable decreased cell proliferation ability in SNORD89 knockdown-OS cells (Fig. 4e). In addition, both plate clone formation assay and soft agar colony formation assay showed that SNORD89 overexpression significantly increased the number of clone formation in OV and CA cells, and the size was larger in OE group (Fig. 4f, g, Additional file 5: Figure S4d, e). Furthermore, colony formation assays indicated that silencing SNORD89 obviously reduced the number of colony formation in OS cells, and the size was smaller in shRNA-1 and shRNA-2 groups (Fig. 4h). These results suggest that SNORD89 may increase cell proliferation and self-renewal ability of ovarian cancer cells.

### Effects of SNORD89 interference on cell migration and invasion of ovarian cancer cells

**Fig. 5:**
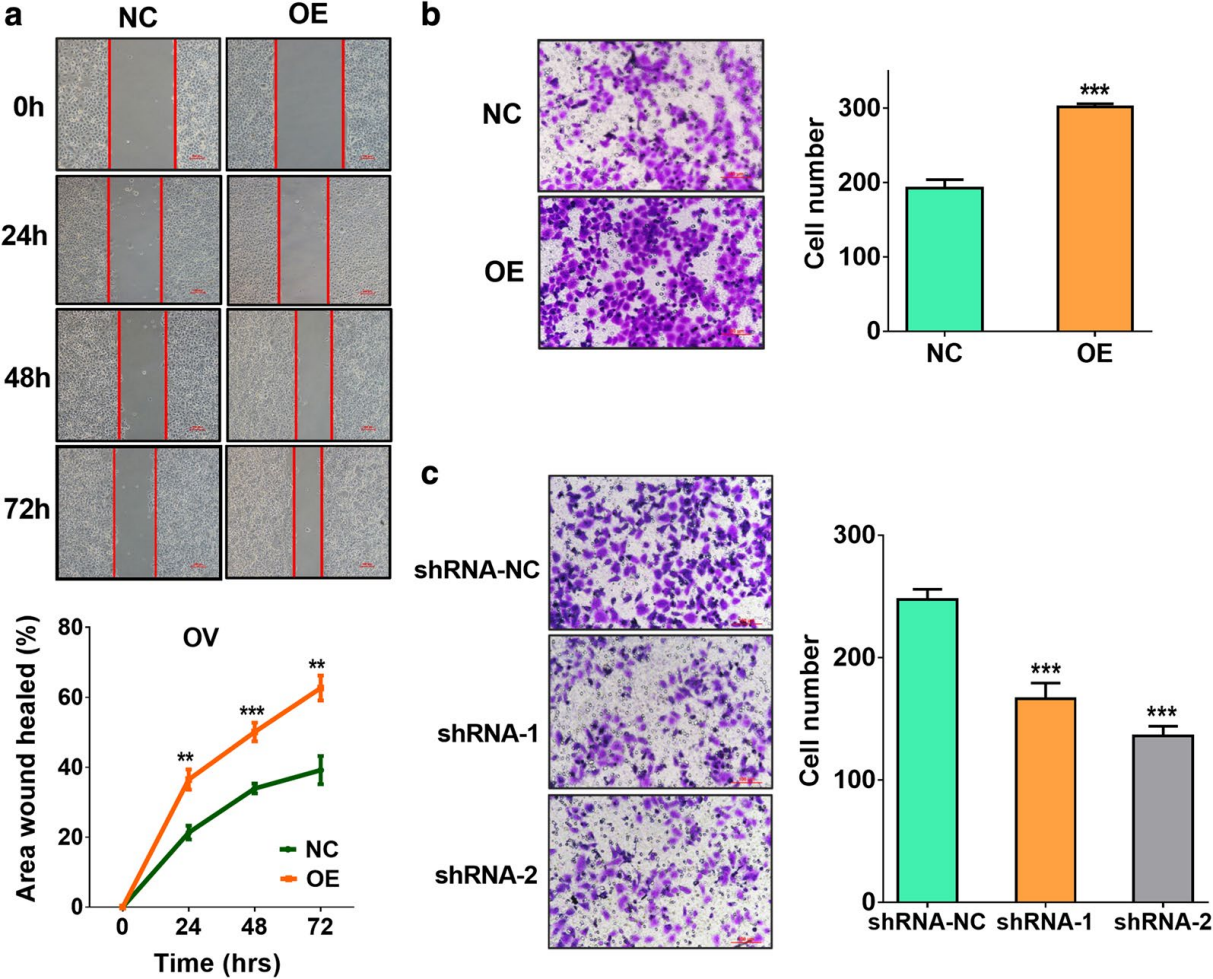
Effects of SNORD89 interference on cell migration and invasion of ovarian cancer cells. **a** The effect of SNORD89 overexpression on the migration ability of ovarian cancer cells by scratch migration assay in OV cells 24 h, 48 h and 72 h after transfection with SNORD89 OE plasmids. **b** The effect of SNORD89 overexpression on the migration ability of ovarian cancer cells by cell invasion analysis in OV cells 48 h after transfection with SNORD89 OE plasmids. **c** The effect of SNORD89 knockdown on the invasion ability of ovarian cancer cells by cell invasion analysis in OS cells 48 h after transfection with shRNA-1 and shRNA-2 plasmids

We further detected the effects of SNORD89 interference on invasion and migration ability of ovarian cancer cells by scratch migration assay and cell invasion analysis. The scratch migration assay showed that the overexpression of SNORD89 significantly increased the area wound healed than NC-transfected OV and CA cells at 24 h, 48 h and 72 h (Fig. 5a and Additional file 5: Figure S4f), suggesting SNORD89 can increase the migration ability of ovarian cancer cells. In addition, the cell invasion analysis indicated that SNORD89 overexpression obviously elevated the invasion ability of OV and CA cells (Fig. 5b and Additional file 5: Figure S4g), while SNORD89 knockdown notably reduced the invasion ability of OS cells (Fig. 5c).

### The carcinogenicity of SNORD89 may be related to the NOTCH1-MYC highway

**Fig. 6:**
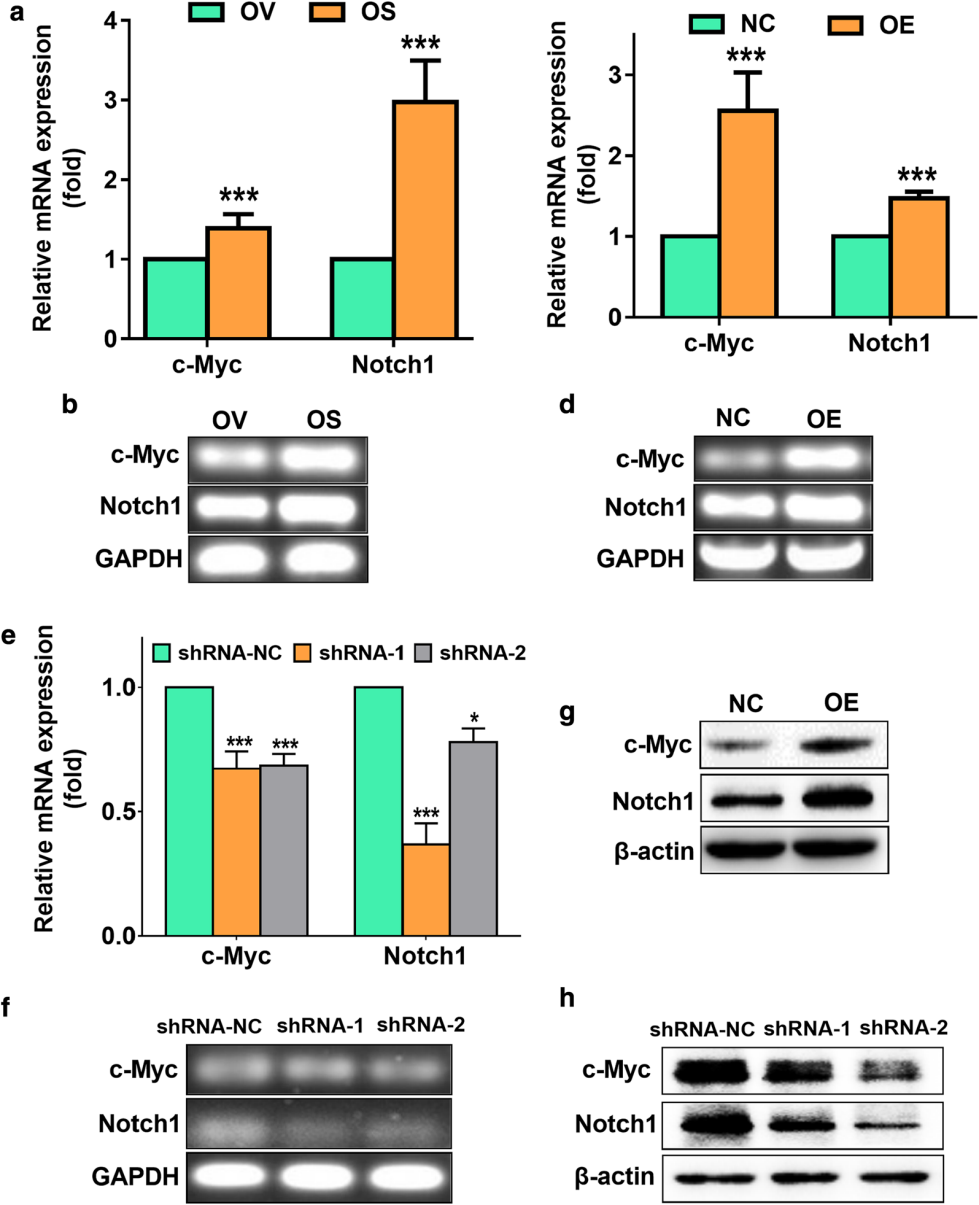
Effects of SNORD89 interference on the expression of c-Myc and Notch1 of ovarian cancer cells. **a** The mRNA expression of c-Myc and Notch1 was detected in OV and OS cells by qRT-PCR. The mRNA expression of the two genes in OV cells was set as 1. **b** The representative agarose gel electrophoresis photos showed the expression of c-Myc and Notch1 in OV and OS cells after RT-PCR. GAPDH was used an endogenous control. **c** The mRNA expression of c-Myc and Notch1 was detected in OV cells transfected with SNORD89 OE or NC plasmids at 24 h by qRT-PCR. The mRNA expression of the two genes in OV cells transfected with NC plasmids was set as 1. **d** The representative agarose gel electrophoresis photos showed the increased expression of c-Myc and Notch1 in OV cells transfected with SNORD89 OE. **e** The mRNA expression of c-Myc and Notch1 was detected in OS cells transfected with shRNA-1, shRNA-2, and shRNA-NC plasmids at 48 h by qRT-PCR. **f** The representative agarose gel electrophoresis photos showed the decreased expression of c-Myc and Notch1 in OS cells transfected with SNORD89 shRNA-1 and shRNA-2. **g** The representative western blot photos showed the increased expression of c-Myc and Notch1 in OV cells transfected with SNORD89 OE. **h** The representative western blot photos showed the decreased expression of c-Myc and Notch1 in OS cells transfected with SNORD89 shRNA-1 and shRNA-2. β-actin was used an endogenous control

Many reports have shown that c-Myc binds with NOTCH1 to promote the development of cancer by acting as a target of NOTCH1 to form a NOTCH1-MYC pathway [24, 25]. We first compared the expression of c-Myc and Notch1 in OV and OS by qRT-PCR, and found that the expression levels of c-Myc and Notch1 were 1.39 ± 0.09 and 2.97 ± 0.37-fold in OS cells than OV cells (Fig. 6a). RT-PCR and agarose gel electrophoresis also verified the increased expression of c-Myc and Notch1 in OS cells (Fig. 6b).

Next, we assessed the effects of SNORD89 interference on the expression of c-Myc and Notch1 in ovarian cancer cells. The Notch1 and c-Myc expression levels were elevated 2.56 ± 0.33 and 1.47 ± 0.05-fold in the OV cells transfected with SNORD89 OE plasmids by qRT-PCR (Fig. 6c), and RT-PCR and agarose gel electrophoresis also showed the consistent results (Fig. 6d). The Notch1 and c-Myc expression levels were elevated 1.75 ± 0.06 and 1.58 ± 0.10-fold in the CA cells transfected with SNORD89 OE plasmids by qRT-PCR (Additional file 5: Figure S4h). Additionally, silencing SNORD89 significantly declined the expression of c-Myc and Notch1 in OS cells (Fig. 6e). The similar results were also verified by RT-PCR and agarose gel electrophoresis (Fig. 6f). We further observed that SNORD89 overexpression obviously increased the protein expression of c-Myc and Notch1 in OV and CA cells (Fig. 6g and Additional file 5: Figure S4i), while SNORD89 knockdown notably decreased the expression of these proteins in OS cells (Fig. 6h).

In our experiments, we found that SNORD89 affected the proliferation, self-renewal ability, invasion and migration of ovarian cancer cells related to stemness phenotype. Notch1 is a well-known pathway associated with stemness phenotype in cancer, and c-Myc can bind with NOTCH1 to promote the development of cancer by acting as a target of NOTCH1 to form a NOTCH1/c-Myc pathway. Thus, the data suggest that SNORD89 might regulate the Notch pathway in ovarian cancer.

## Discussion

Ovarian cancer is the most lethal of the women tumors, because it is rarely diagnosed at an early stage and most patients are diagnosed at the advanced stage. Therefore, ovarian cancer is historically called the “silent killer” [26]. Though ovarian cancer is sensitive to chemotherapeutic drugs, about 60% of the advanced ovarian cancer patients will ultimately recue within 5 years, and some will develop resistance [27–30]. Thus, the key to the successful treatment of ovarian cancer is its early detection.

Small nucleolar RNA (SnoRNA) is a kind of single-stranded small molecule non-coding RNA widely distributed in nucleolus of eukaryotic cells, with stable metabolism and a length of 60–300 nucleotide sequences [7, 31]. They interact with ribonucleoproteins to form stable small nucleolar ribonucleoproteins particles (snoRNPs), which are involved in the post-transcriptional modification of ribosomal RNA and other RNAs [32, 33]. SnoRA42 was reported to have an important role in lung tumorigenesis as an oncogene [34, 35]. Xu et al. demonstrated that SNORND113-1 functioned as a tumor suppressor in hepatocellular carcinoma (HCC) [36]. However, few studies on SNORNAs in ovarian cancer were carried out at present. In the present study, we identified 4 SNORNAs associated with the poor prognosis of ovarian cancer patients from TCGA database. Among them, the high expression of snord116-4 and SNORD89 was associated with patients’ poor prognosis. Further analysis revealed that the expression of SNORD89 was higher in elderly patients and progressive disease patients, and SNORD89 high expression reversed the survival curve of stage III in ovarian cancer patients, suggesting the main role of SNORD89 in the progress of ovarian cancer.

Cancer stem cells (CSCs) are a small group of dormant cells that have self-renewal, infinite proliferation, invasion and migration abilities, which can lead to the recurrence and metastasis of tumors after conventional therapy [23, 37, 38]. In recent years, ovarian cancer has been described as a kind of stem cell disease [39]. Here, we obtained OVCAR-3 (OV) sphere-forming (OS) cells with higher expression of stem cell markers, CD133, CD44, and Nanog, by culturing OVCAR-3 (OV) cells in suspension in serum-free medium. We found that SNORD89 highly expressed in OS than OV and HOSEpiC by gene chips and qRT-PCR analysis. Furthermore, overexpression of SNORD89 upregulated the expression of Nanog, CD44 and CD133, and increased the cell proliferation and self-renewal ability of OV and CA cells. Conversely, silencing SNORD89 resulted in the downregulation of Nanog, CD44 and CD133 expression, and the decreased cell proliferation and self-renewal ability of OS cells. The results showed the main roles of SNORD89 in the stemness regulation of ovarian cancer cells. This is the first report of SNORNAs in regulating stemness of ovarian cancer. SNORD78 was reported to be required for the self-renewal of cancer-stem cells of non-small cell lung cancer (NSCLC) [12]. Mannoor et al. also revealed that SNORA42 had important influences in regulating features of lung tumor-initiating cells (TICs) [21].

Additionally, we found the expression level of SNORD89 was related to some genes of cell cycle checkpoint and cell cycle arrest by analyzing TCGA database. Furthermore, SNORD89 overexpression upregulated the proportion of S phase of OV cells, whereas SNORD89 silence downregulated the proportion of S phase of OS cells. There are some other SNORNAs that were reported to regulate cell cycle and proliferation of tumor cells. SNORD78 knockdown could inhibit the proliferation of NSCLC cells via inducing cell cycle arrest at G0/G1 phase [12]. Valleron et al. demonstrated that SNORD114-1 variant could regulate G0/G1 to S phase transition to promote cell growth by the Rb/p16 pathways in acute leukemia [40]. Our study revealed that SNORD89 might modulate cell cycle to promote proliferation of ovarian cancer cells by regulating stemness.

Besides, scratch migration assay and trans-well invasion analysis showed that SNORD89 could promote the migration and invasion ability of ovarian cancer cells in our study. Cui et al. reported that SNORA23 knockdown decreased the invasive potential of Pancreatic Ductal Adenocarcinoma (PDAC) cells [19]. Crea et al. found that SNORA55 silencing inhibited cell migration in prostate cancer cell lines [41]. Our study suggests that SNORD89 has a potential influence on the progression of ovarian cancer by promoting cell migration and invasion.

NOTCH signaling is involved in the regulation of cancer and stem cells. NOTCH1-4 receptors have complex functions in different tissues and tumors. NOTCH1 signaling could regulate self-renewal and resistance of CSCs [24]. C-Myc is an important direct target of Notch1 in various cancers, such as T-cell lymphoblastic leukemias [25, 42, 43], breast cancer [44, 45], lung adenomas and head [46] and neck squamous cell carcinoma [47]. We found that c-Myc and Notch1 highly expressed in OS cells compared with the parental OV cells. SNORD89 overexpression significantly increased the expression of c-Myc and Notch1 in mRNA and protein levels in OV and CA cells, whereas SNORD89 knockdown notably decreased their expression in OS cells. After the overexpression of SNORD89, the expression levels of c-Myc and Notch1 was increased. The number of combinations of c-Myc and NOTCH1 will raise as the increased expression and then promoting the stemness phenotype and development of ovarian cancer. Therefore, we speculate that the oncogenic effect of SNORD89 may be related to the regulation of Notch1-c-Myc pathway.

## Conclusion

In conclusion, we demonstrate that the expression of SNORD89 was associated with the prognosis of ovarian cancer patients and involved in the stemness regulation of ovarian cancer cells. SNORD89 could influence the stemness of ovarian cancer cells to promote cell proliferation, self-renewal, and invasion of ovarian cancer cells. The stemness regulation of SNORD89 might be mediated by the activation of Notch1-c-Myc pathway. Our study will supply an important clue that SNORD89 may facilitate the development of SNORNA-directed diagnostics and therapeutics against ovarian cancer.

## Additional files

**Additional file 1: Figure S1.** Survival analysis of SNORD89 and SNORD116-4 in different stages of ovarian cancer patients of TCGA. Kaplan–Meier survival curves for OS in (a) stage III and (b) stage IV of ovarian cancer patients based on the expression of SNORD89. Kaplan–Meier survival curves for OS in (c) stage III and (d) stage IV of ovarian cancer patients based on the expression of SNORD116-4. e Kaplan–Meier survival curves for OS in ovarian cancer patients based on stage III and stage IV. f Kaplan–Meier survival curves for OS in stage III, stage IV, SNORD89 low and SNORD89 high of ovarian cancer patients. Cut off threshold was median value in each cohort.

**Additional file 2: Figure S2.** 5-year survival analysis of SNORD89 and SNORD116-4 in different stages of ovarian cancer patients of TCGA. Kaplan–Meier survival curves for OS in (a) stage III and (b) stage IV of ovarian cancer patients based on the expression of SNORD89. Kaplan–Meier survival curves for OS in (c) stage III and (d) stage IV of ovarian cancer patients based on the expression of SNORD116-4. e Kaplan–Meier survival curves for OS in ovarian cancer patients based on stage III and stage IV. f Kaplan–Meier survival curves for OS in stage III, stage IV, SNORD89 low and SNORD89 high of ovarian cancer patients. Cut off threshold was median value in each cohort.

**Additional file 3: Table S1.** Correlation between SNORNA89 and SNORD116-4 expression and the clinicopathologic features of ovarian cancer patients in TCGA (Chi square test). **Table S2.** Univariate and multivariate analysis for predictors of overall survival (OS) of ovarian cancer patients in TCGA. **Table S3.** Univariate and multivariate analysis for predictors of progression-free survival (PFS) of ovarian cancer patients in TCGA. **Table S4.** 15 dysregulation snoRNAs in HOSEpiC, OV and OS cells.

**Additional file 4: Figure S3.** Correlation between SNORD89 expression and the clinicopathologic features of ovarian cancer patients (unpaired t test). a The comparison of SNORD89 expression in different ages of ovarian cancer patients. b The comparison of SNORD89 expression in different therapy outcome of ovarian cancer patients.

**Additional file 5: Figure S4.** Effects of SNORD89 interference on biological behaviors in CAOV-3 cells. a The SNORD89 expression in CA cells transfected with over expression (OE) of SNORD89 plasmid or negative control (NC) plasmid at 24, 48, and 72 h by qRT-PCR. The SNORD89 expression in CA cells transfected with NC plasmids was set as 1. b The mRNA expression of CD133, CD44 and Nanog were detected in CA cells transfected with SNORD89 OE or NC plasmids at 24 h by qRT-PCR. The mRNA expression of these genes in CA cells transfected with NC plasmids was set as 1. c The cell proliferation was measured in CA cells of SNORD89 overexpression by Cell Counting Kit-8 (CCK-8) assays at 24 h, 48 h, 72 h and 96 h transfection. d The cell proliferation ability was measured in CA cells of SNORD89 overexpression by plate clone formation assay. e The cell self-renewal ability was measured in CA cells of SNORD89 overexpression by soft agar colony formation assay. f The effect of SNORD89 overexpression on the migration ability of ovarian cancer cells by scratch migration assay in CA cells 24 h, 48 h and 72 h after transfection with SNORD89 OE plasmids. g The effect of SNORD89 overexpression on the migration ability of ovarian cancer cells by cell invasion analysis in CA cells 48 h after transfection with SNORD89 OE plasmids. h The mRNA expression of c-Myc and Notch1 was detected in CA cells transfected with SNORD89 OE or NC plasmids at 24 h by qRT-PCR. The mRNA expression of the two genes in CA cells transfected with NC plasmids was set as 1. i The representative western blot photos showed the increased expression of c-Myc and Notch1 in CA cells transfected with SNORD89 OE.

## Data Availability

The datasets analyzed during the current study are available in the TCGA repository, https://cancergenome.nih.gov/.

## Abbreviations

CSCs: cancer stem cells
SnoRNA: nucleolar small molecule RNA
snoRNPs: small nucleolar ribonucleoproteins particles
rRNA: ribosomal RNA
TICs: tumor-initiating cells
AML: acute myelogenous leukaemia
NC RNAs: non-coding RNAs
HOSEpiC: ovarian epithelial cells
OV: ovarian cancer cells
CA: CAOV-3
OS: ovarian cancer stem cells
qRT-PCR: quantitative real-time PCR
RT-PCR: reverse transcription PCR
OE: over expression plasmid
shRNA: silent plasmid
NC: negative control
shRNA-NC: shRNA negative control
CCK-8: Cell Counting Kit-8
snRNAs: small nuclear RNA
HCC: hepatocellular carcinoma
CSCs: cancer stem cells
NSCLC: non-small cell lung cancer
PDAC: Pancreatic Ductal Adenocarcinoma
