# Supplementary figures and images for: SNORD89 promotes stemness phenotype of ovarian cancer cells by regulating Notch1-c-Myc pathway
Source: J Transl Med. 2019 Aug 8;17:259. doi: 10.1186/s12967-019-2005-1 (PMC6686521; doi:10.1186/s12967-019-2005-1)

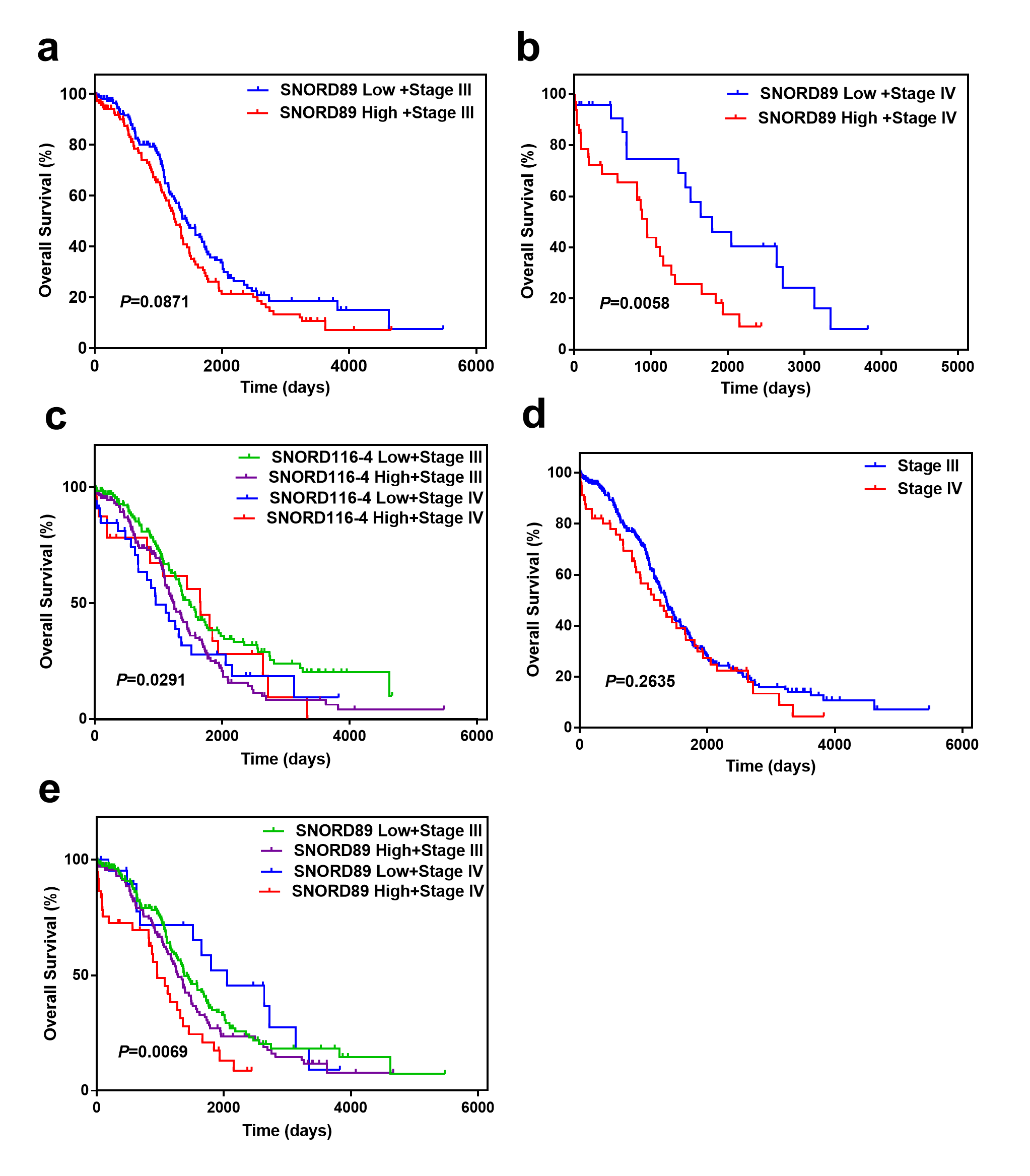

Supplement: Supplementary file 1 — Additional file 1: Figure S1. Survival analysis of SNORD89 and SNORD116-4 in different stages of ovarian cancer patients of TCGA. Kaplan–Meier survival curves for OS in (a) stage III and (b) stage IV of ovarian cancer patients based on the expression of SNORD89. Kaplan–Meier survival curves for OS in (c) stage III and (d) stage IV of ovarian cancer patients based on the expression of SNORD116-4. e Kaplan–Meier survival curves for OS in ovarian cancer patients based on stage III and stage IV. f Kaplan–Meier survival curves for OS in stage III, stage IV, SNORD89 low and SNORD89 high of ovarian cancer patients. Cut off threshold was median value in each cohort. [file 12967_2019_2005_MOESM1_ESM.tif]

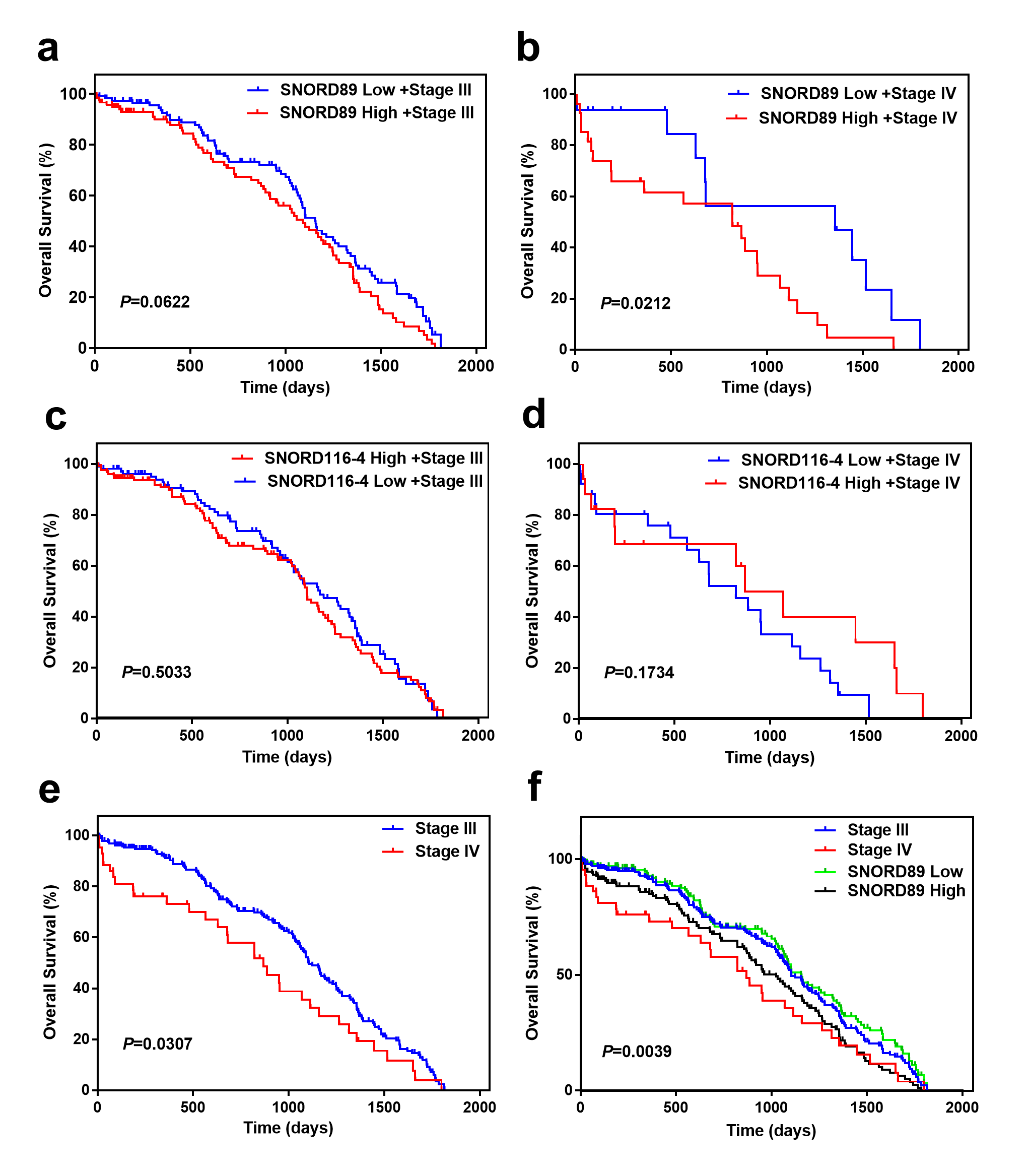

Supplement: Supplementary file 2 — Additional file 2: Figure S2. 5-year survival analysis of SNORD89 and SNORD116-4 in different stages of ovarian cancer patients of TCGA. Kaplan–Meier survival curves for OS in (a) stage III and (b) stage IV of ovarian cancer patients based on the expression of SNORD89. Kaplan–Meier survival curves for OS in (c) stage III and (d) stage IV of ovarian cancer patients based on the expression of SNORD116-4. e Kaplan–Meier survival curves for OS in ovarian cancer patients based on stage III and stage IV. f Kaplan–Meier survival curves for OS in stage III, stage IV, SNORD89 low and SNORD89 high of ovarian cancer patients. Cut off threshold was median value in each cohort. [file 12967_2019_2005_MOESM2_ESM.tif]

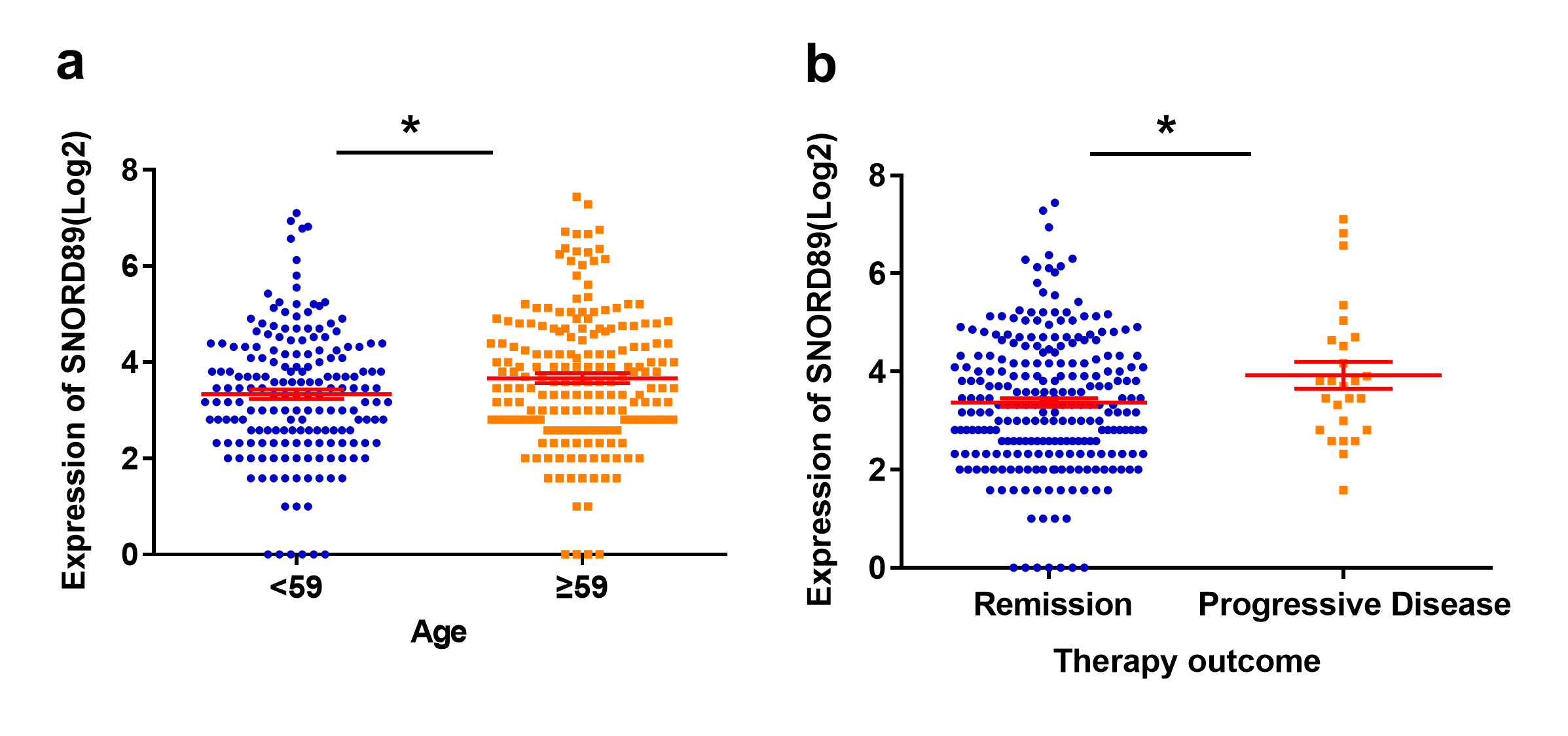

Supplement: Supplementary file 4 — Additional file 4: Figure S3. Correlation between SNORD89 expression and the clinicopathologic features of ovarian cancer patients (unpaired t test). a The comparison of SNORD89 expression in different ages of ovarian cancer patients. b The comparison of SNORD89 expression in different therapy outcome of ovarian cancer patients. [file 12967_2019_2005_MOESM4_ESM.tif]

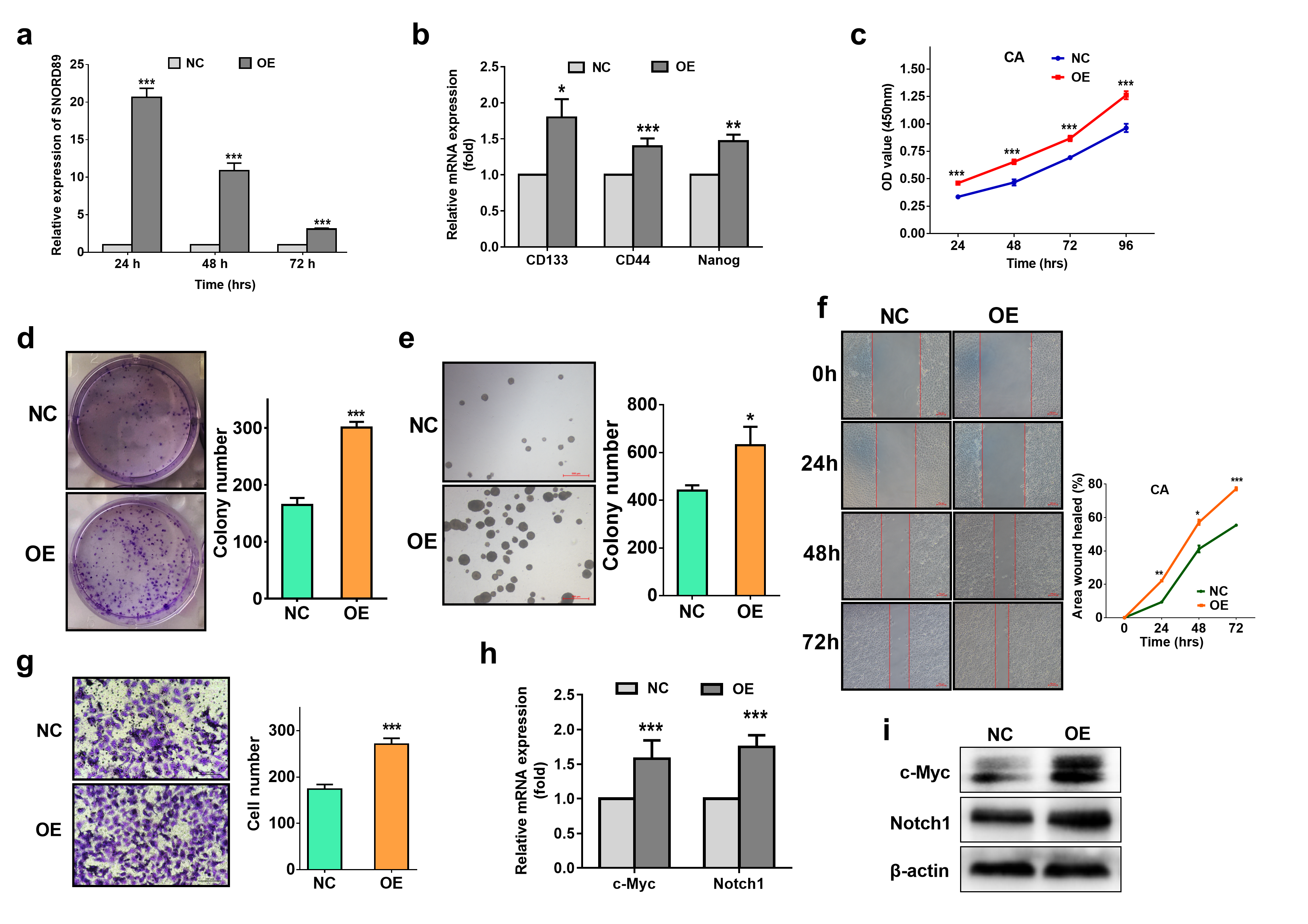

Supplement: Supplementary file 5 — Additional file 5: Figure S4. Effects of SNORD89 interference on biological behaviors in CAOV-3 cells. a The SNORD89 expression in CA cells transfected with over expression (OE) of SNORD89 plasmid or negative control (NC) plasmid at 24, 48, and 72 h by qRT-PCR. The SNORD89 expression in CA cells transfected with NC plasmids was set as 1. b The mRNA expression of CD133, CD44 and Nanog were detected in CA cells transfected with SNORD89 OE or NC plasmids at 24 h by qRT-PCR. The mRNA expression of these genes in CA cells transfected with NC plasmids was set as 1. c The cell proliferation was measured in CA cells of SNORD89 overexpression by Cell Counting Kit-8 (CCK-8) assays at 24 h, 48 h, 72 h and 96 h transfection. d The cell proliferation ability was measured in CA cells of SNORD89 overexpression by plate clone formation assay. e The cell self-renewal ability was measured in CA cells of SNORD89 overexpression by soft agar colony formation assay. f The effect of SNORD89 overexpression on the migration ability of ovarian cancer cells by scratch migration assay in CA cells 24 h, 48 h and 72 h after transfection with SNORD89 OE plasmids. g The effect of SNORD89 overexpression on the migration ability of ovarian cancer cells by cell invasion analysis in CA cells 48 h after transfection with SNORD89 OE plasmids. h The mRNA expression of c-Myc and Notch1 was detected in CA cells transfected with SNORD89 OE or NC plasmids at 24 h by qRT-PCR. The mRNA expression of the two genes in CA cells transfected with NC plasmids was set as 1. i The representative western blot photos showed the increased expression of c-Myc and Notch1 in CA cells transfected with SNORD89 OE. [file 12967_2019_2005_MOESM5_ESM.tif]
