# Supplementary material for: SNORD89 promotes stemness phenotype of ovarian cancer cells by regulating Notch1-c-Myc pathway
Source: J Transl Med. 2019 Aug 8;17:259. doi: 10.1186/s12967-019-2005-1 (PMC6686521; doi:10.1186/s12967-019-2005-1)
Supplement: Supplementary file 3 — Additional file 3: Table S1. Correlation between SNORNA89 and SNORD116-4 expression and the clinicopathologic features of ovarian cancer patients in TCGA (Chi square test). Table S2. Univariate and multivariate analysis for predictors of overall survival (OS) of ovarian cancer patients in TCGA. Table S3. Univariate and multivariate analysis for predictors of progression-free survival (PFS) of ovarian cancer patients in TCGA. Table S4. 15 dysregulation snoRNAs in HOSEpiC, OV and OS cells. [file 12967_2019_2005_MOESM3_ESM.docx]

**Table S1. Correlation between SNORNA89 and SNORD116-4 expression and the clinicopathologic features of ovarian cancer patients in TCGA (Chi square test).**

|  | SNORD89 expression | | |  | SNORD116-4 expression | | |
| --- | --- | --- | --- | --- | --- | --- | --- |
| Variable | Low（<9.4） | High  (≥9.4) | *P* value |  | Low  （<5.9） | High  (≥5.9) | *P* value |
| Age |  |  | **0.03** |  |  |  | 0.259 |
| ＜59 | 103 | 82 |  |  | 95 | 91 |  |
| ≥59 | 84 | 105 |  |  | 86 | 104 |  |
| Subdivision |  |  | 0.92 |  |  |  | 0.511 |
| left or right | 50 | 50 |  |  | 46 | 55 |  |
| Bilateral | 125 | 128 |  |  | 125 | 128 |  |
| Lymphatic invasion |  |  | 0.583 |  |  |  | 0.578 |
| NO | 20 | 28 |  |  | 22 | 26 |  |
| YES | 46 | 53 |  |  | 41 | 59 |  |
| Grade |  |  | 0.841 |  |  |  | 0.395 |
| G1‎+G2 | 21 | 22 |  |  | 19 | 24 |  |
| G3+G4 | 162 | 159 |  |  | 164 | 157 |  |
| Race |  |  | 0.407 |  |  |  | 0.117 |
| Asian | 4 | 7 |  |  | 7 | 4 |  |
| Black or African American | 15 | 10 |  |  | 8 | 17 |  |
| White | 163 | 162 |  |  | 167 | 158 |  |
| Stage |  |  | 0.475 |  |  |  | 0.192 |
| Stage3 | 149 | 142 |  |  | 141 | 150 |  |
| stage4 | 24 | 33 |  |  | 33 | 24 |  |
| Tumor Size |  |  | 0.713 |  |  |  | 0.488 |
| ≤20mm | 130 | 132 |  |  | 132 | 130 |  |
| ＞20mm | 33 | 37 |  |  | 32 | 38 |  |
| Venous invasion |  |  | 0.151 |  |  |  | 0.991 |
| NO | 19 | 21 |  |  | 21 | 19 |  |
| YES | 39 | 24 |  |  | 33 | 30 |  |
| Therapy outcome |  |  | 0.326 |  |  |  | 0.191 |
| Remission | 137 | 115 |  |  | 136 | 116 |  |
| Progressive disease | 12 | 15 |  |  | 11 | 16 |  |

**Table S2.** **Univariate and multivariate analysis for predictors of overall survival (OS) of ovarian cancer patients in TCGA**

| Factor |  | Univariate analysis | | |  | Multivariate analysis | | |
| --- | --- | --- | --- | --- | --- | --- | --- | --- |
|  |  | HR | 95%CI | *P* |  | HR | 95%CI | *P* |
| SNORD89 Expression | Low | 1.402 | 1.081-1.818 | **0.011** |  | 1.294 | 0.475-3.524 | 0.614 |
|  | High |  |  |  |  |  |  |  |
| Age |  | 1.020 | 1.008-1.033 | **0.001** |  | 0.987 | 0.948-1.026 | 0.501 |
| lymphatic invasion | YES | 1.420 | 0.837-2.408 | 0.193 |  | 3.987 | 0.997-15.947 | **0.051** |
|  | NO |  |  |  |  |  |  |  |
| Race | ASIAN | 0.733 | 0.519-1.036 | 0.079 |  | 0.410 | 0.188-0.891 | **0.024** |
|  | BLACK |  |  |  |  |  |  |  |
|  | WHITE |  |  |  |  |  |  |  |
| Stage | Stage 3 | 1.213 | 0.864-1.704 | 0.265 |  | 0.718 | 0.258-1.999 | **0.526** |
|  | Stage 4 |  |  |  |  |  |  |  |
| Tumor Size | ≤20mm | 1.632 | 1.188-2.242 | **0.002** |  | 1.480 | 0.549-3.990 | 0.438 |
|  | >20 mm |  |  |  |  |  |  |  |
| Venous invasion | YES | 0.905 | 0.487-1.683 | 0.192 |  | 0.179 | 0.040-0.801 | **0.024** |
|  | NO |  |  |  |  |  |  |  |

**Table S3. Univariate and multivariate analysis for predictors of progression-free survival (PFS) of ovarian cancer patients** **in TCGA**

| Factor |  | Univariate analysis | | |  | Multivariate analysis | | |
| --- | --- | --- | --- | --- | --- | --- | --- | --- |
|  |  | HR | 95%CI | *P* |  | HR | 95%CI | *P* |
| SNORD89 Expression | Low | 1.234 | 0.916-1.663 | 0.166 |  | 0.596 | 0.168-2.116 | 0.423 |
|  | High |  |  |  |  |  |  |  |
| Age |  | 1.019 | 1.005-1.033 | **0.007** |  | 1.023 | 0.969-1.080 | 0.407 |
| lymphatic invasion | YES | 1.336 | 0.725-2.463 | 0.353 |  | 2.579 | 0.401-16.564 | 0.318 |
|  | NO |  |  |  |  |  |  |  |
| Race | ASIAN | 1.048 | 0.709-1.548 | 0.814 |  | 0.531 | 0.192-1.475 | 0.225 |
|  | BLACK |  |  |  |  |  |  |  |
|  | WHITE |  |  |  |  |  |  |  |
| Stage | Stage 3 | 1.216 | 0.819-1.804 | 0.332 |  | 1.190 | 0.345-4.107 | 0.783 |
|  | Stage 4 |  |  |  |  |  |  |  |
| Tumor Size | ≤20mm | 1.065 | 0.722-1.569 | 0.752 |  | 1.182 | 0.306-4.570 | 0.808 |
|  | >20 mm |  |  |  |  |  |  |  |
| Venous invasion | YES | 0.526 | 0.252-1.099 | 0.087 |  | 0.114 | 0.017-0.775 | **0.026** |
|  | NO |  |  |  |  |  |  |  |

**Table S4.** **15 dysregulation snoRNAs in HOSEpiC, OV and OS cells.**

|  | [OS] vs [HOSEpiC] | | [OS] vs [OV] | | [HOSEpiC] vs [OV] | | |
| --- | --- | --- | --- | --- | --- | --- | --- |
| Gene Symbol | Regulation | FC | Regulation | FC | Regulation | | FC |
| **SNORD89** | up | 3.366167 | up | 3.5164409 | up | 1.044642438 | |
| SNORD69 | up | 6.083523 | up | 16.004517 | up | 2.630797484 | |
| SNORA38B | up | 2.0316525 | up | 16.920288 | up | 8.328337646 | |
| SNORA74B | up | 6.9150314 | up | 24.40511 | up | 3.529284046 | |
| SNORA52 | up | 4.1898546 | up | 24.663404 | up | 5.886458208 | |
| SNORA45 | up | 2.2137408 | up | 25.007618 | up | 11.29654294 | |
| SNORD53 | up | 4.1239567 | up | 25.54687 | up | 6.194747389 | |
| SNORD83A | up | 3.290171 | up | 34.693256 | up | 10.54451456 | |
| SNORA46 | up | 2.4330056 | up | 40.279503 | up | 16.55545018 | |
| SNORA71B | up | 2.1214023 | up | 47.181244 | up | 22.24059246 | |
| SNORD14D | up | 11.178123 | up | 92.269226 | up | 8.254447191 | |
| SNORA42 | up | 22.996056 | up | 130.92723 | up | 5.693464566 | |
| SNORD14E | up | 4.37487 | up | 191.50165 | up | 43.7731064 | |
| SNORA38 | up | 6.0100484 | up | 204.01624 | up | 33.94585641 | |
| SNORA72 | up | 13.796497 | up | 279.55872 | up | 20.26302184 | |
